# Bladder function in children with posterior urethral valves: impact of antenatal versus postnatal diagnosis

**DOI:** 10.1590/S1677-5538.IBJU.2021.0046

**Published:** 2021-09-14

**Authors:** Osama M. Sarhan, Bassem S. Wadie, Fouad Al-Kawai, Mohamed Dawaba

**Affiliations:** 1 Mansoura University Faculty of Medicine Urology and Nephrology Center Mansoura Egypt Urology and Nephrology Center, Faculty of Medicine, Mansoura University, Mansoura, Egypt; 2 King Fahad Specialist Hospital Department of Urology Dammam Saudi Arabia Department of Urology, King Fahad Specialist Hospital, Dammam, Saudi Arabia

**Keywords:** Urinary Bladder, Overactive, Postnatal Care, Urethra

## Abstract

**Purpose:**

Posterior urethral valves (PUVs) are the most common cause of congenital bladder obstruction in boys. Our aim was to assess the impact of early diagnosis and fulguration of PUVs on bladder function and compare their functional and urodynamic outcome with children who underwent delayed intervention.

**Materials and Methods:**

We retrospectively evaluated 153 patients who underwent primary valve ablation from two tertiary hospitals between 2001 and 2018. Patients have been divided into 2 groups, group 1 included 69 patients who were detected antenatally and underwent early fulguration of PUVs while group 2 included 84 children presented postnatally and underwent delayed valve ablation. The recorded data throughout follow-up in renal function tests, urodynamics and changes in the upper urinary tracts were evaluated and compared.

**Results:**

Median age at time of valve ablation was 10 days in group 1 and 7 months in group 2. The median follow-up period was 6.5 and 7 years in group 1 and 2, respectively. Chronic kidney disease (CKD) developed in 15 (22%) boys in group 1 while in group 2 it was observed in 31 (37%), p=0.04. While Q-max, mean bladder capacity and post-void residual (PVR) volumes were comparable in both groups, percent PVR was significantly higher in group 2 (3.27 vs. 1.44, p=0.002). Detrusor overactivity was slightly different in both groups (p = 0.07).

**Conclusions:**

Compared to delayed intervention, primary ablation of PUVs during the early neonatal life possibly provides the optimum chance to have optimum renal function without impact on bladder function.

## INTRODUCTION

Posterior urethral valves (PUVs) are the most common cause of lower urinary tract obstruction in male neonates (1). With routine use of antenatal ultrasound screening, most cases are now detected antenatally, which allows early intervention (2, 3). Some cases can be missed during antenatal screening and present after birth with a wide range of symptoms. The functional impact of PUVs on the lower urinary tract ranges from mild bladder dysfunction to urinary incontinence, and the latter was found in over 20% of children 1 year after valve ablation (4).

Voiding dysfunction was reported in up to 75% of boys with PUVs (5). It originates in utero, after infravesical obstruction brought about by the valve and usually remains during infancy and through adolescence, even after valve ablation (6, 7). Impairment of kidney function is sometimes progressive, and despite successful valve ablation, approximately one third of PUVs patients develop chronic kidney disease (CKD) (1, 3, 5).

Primary valve ablation is the gold standard for early postnatal management of PUVs (8). Different studies have been published to assess the functional and histologic changes taking place in the valve bladder (6, 7, 9). The value of urodynamic evaluation in PUVs management has been previously emphasized. Glassberg suggested that valve ablation alone without urodynamic follow-up is not appropriate (9). Instead, others preferred non-invasive methods for follow-up like bladder diary, uroflowmetry and post-void residual volume measurements (10).

In childhood, the bladder function is often compromised in patients with PUVs. In infancy abnormal bladder function is characterized by low compliance or overactivity, but later the bladder becomes oversized with poor emptying (7, 11, 12). Polyuria, which is often associated with renal failure and secondary changes in the bladder neck impacts bladder function (13). Toilet training is often delayed in children with PUVs. Patients with PUVs achieve daytime and nighttime urinary continence significantly later than their healthy peers (14). Neonatal valve ablation was reported to protect the bladder and allow normal cycling, which helps in bladder healing and gives a chance for the bladder to revert to a normal voiding pattern and avoid voiding dysfunction (15).

In this study, we tried to show whether antenatal diagnosis and early valve ablation better serve bladder function in boys with PUVs. We studied two groups of children from two different centers; one group had antenatal diagnosis and early fulguration, while in the other diagnosis was made after the disease becomes symptomatic during infancy and childhood with a delayed valve ablation.

It was hypothesized that antenatal diagnosis and early valve ablation improves renal function and upper tract changes in PUVs patients. We decided to evaluate the effect of antenatal diagnosis and early PUVs ablation on the future bladder function and lower urinary tract changes in those patients and to compare their functional and urodynamic outcome with children who underwent delayed intervention.

## PATIENTS AND METHODS

After obtaining the institutional review board approval from the two tertiary hospitals (R.18.05.42 and 0127), records of all patients who underwent PUVs ablation between 2001 and 2018 were reviewed. We included only patients who underwent primary valve ablation for PUVs while patients who underwent urinary diversion (ureterostomy or vesicostomy) and patients with incomplete files were excluded from the study.

Patients were allocated according to the mode of diagnosis into two groups: group 1 comprised 69 children in whom antenatal diagnosis was the rule, while group 2 included 84 children presenting postnatally complaining from symptoms suggestive of valve disease e.g., urinary retention, recurrent UTI, difficulty on micturition, etc. Diagnosis of PUVs was confirmed in all patients by a voiding cystourethrogram (VCUG) which showed ballooning of the posterior urethra as the mainstay of diagnosis of PUVs. At diagnosis, VUR was detected in 37/69 patients (53%) in group 1 and in 41/84 patients (49%) in group 2, respectively.

All children had primary valve ablation using a cold knife urethrotome with a median age at valve ablation of 10 days (IQR, 6-18) and 7 months (IQR, 3-19) for group 1 and 2, respectively. After valve ablation was done, a regular follow-up with clinical examination, serum creatinine, urinalysis, and renal US and VCUG were performed at intervals. Renal function was assessed by determination of serum creatinine levels and the estimation of the glomerular filtration rate (eGFR) by the Schwartz formula, height in cm x K/serum creatinine, where K=0.45 for infants, K=0.55 for patients aged 1 to 13 years and K=0.7 for those aged 13 to 21 years (16). The stage of CKD was determined according to recommendations from the National Kidney Foundation (17). Chronic kidney disease (CKD) was defined as an eGFR <60mL/min/1.73m^2^ and end-stage renal disease (ESRD) was defined as an eGFR <15mL/min/1.73m^2^ or the need for renal replacement therapy.

Bladder functions were studied in both groups by clinical history and voiding diaries uroflowmetry, and filling cystometry. The attainment of urinary continence was recorded. Urinary continence was defined as being totally dry during both day and night with no need for diapers. Periodic follow-up was performed to assess voiding dysfunction, including self-voiding and urinary incontinence.

Urodynamic evaluation was necessary for patients who had persistent or progressive upper tract dilatation after successful valve ablation with lower urinary tract symptoms. Children having persistent symptoms like hesitancy, straining or weak stream, intermittency, dysuria, diurnal dribbling, evidence of incomplete voiding on bladder scan with significant post voiding residual urine (PVR) or urinary incontinence were scheduled for urodynamics. Urodynamic study was carried out in a standard protocol. A 6 or 8 French catheter was used for measuring bladder pressure and infusion of distilled water at room temperature. A rectal catheter was used for abdominal pressure recording. Postvoiding residual urine (PVR) was measured once the catheter is inserted within 5 minutes of the child's last void. The rate of infusion was adjusted at 10^th^ the expected capacity for the age. Voiding was allowed in the urodynamic sitting. For those who could not void with a catheter in place, free uroflowmetry was allowed, using a weight transducer flowmeter.

Bladder dysfunction was categorized as unstable, low compliance, or myogenic failure. Detrusor overactivity occurred when there were uninhibited detrusor contractions of >15cm H2O. Low compliance occurred when the detrusor pressure progressively increased during bladder filling and the difference between the initial and final pressures was >15cm H2O. Bladder compliance was graded as severely impaired if less than 10mL/cm water, moderately impaired if 10-20mL/cm water, mildly impaired if 21-30mL/cm water, and normal if above 30mL/cm water (18).

Expected bladder capacity for age was determined using the formula: capacity in mL=(age in years+2) × 30 for children 2 to 11 years old (19). In children older than 11 years’ normal capacity for adults was used. Decreased bladder capacity was assessed as a reduction of >65% of bladder volume identified based on a voiding diary or estimated bladder capacity in uroflowmetry analyses. Ratio of the actual capacity/expected capacity for age and the ratio of PVR to 15% capacity were calculated for both groups. Myogenic failure was considered when the bladder capacity was larger than that expected for age and was associated with a maximum detrusor pressure during voiding of <20cmH2O and PVR >15% of bladder capacity.

Data were collected, tabulated, and processed using SPSS, version 20 (SPSS Inc., Chicago, IL, USA). For quantitative data, the median and inter-quartile range (IQR) or mean and standard deviation (SD) were calculated when appropriate. The recorded data throughout follow-up in renal function tests, urodynamics changes were evaluated and compared. For categorical variables, Chi square test was executed for inter-groups comparison while, for independent variables; Levene's test and Mann-Whitney U test were used for inter-groups comparison. A p value <0.05 was significant.

## RESULTS

Group 1 included 69 boys who have been diagnosed antenatally with a median gestational age at diagnosis of 31 weeks (IQR, 27-34). Group 2 comprised 84 boys who presented postnatally with a median age at diagnosis of 6 months (IQR, 2-18). The commonest presentation in group 2 was urinary retention in 47, urinary tract infection in 16, difficulty and dysuria in 13 and enuresis in 8 patients.

The follow-up period ranged from 2 to 15 years with a median follow-up of 6.5 years (IQR, 3-9) and it was comparable between both groups. Initial mean serum creatinine was 1.06 vs. 1.15mg/dL, and the final mean serum creatinine was 0.92 vs. 1.45mg/dL in groups 1 and 2, respectively. The difference between means was not statistically significant initially, but the final mean serum creatinine differs significantly between the two groups. At the last follow-up, 46 patients developed CKD (30%); 15 in group 1 versus 31 in group 2, respectively, and the difference was statistically significant (p=0.04). The incidence of VUR at last VCUG was 23% and 27% in group 1 and 2, respectively, and the difference was not statistically significant.

**Figure 1:**
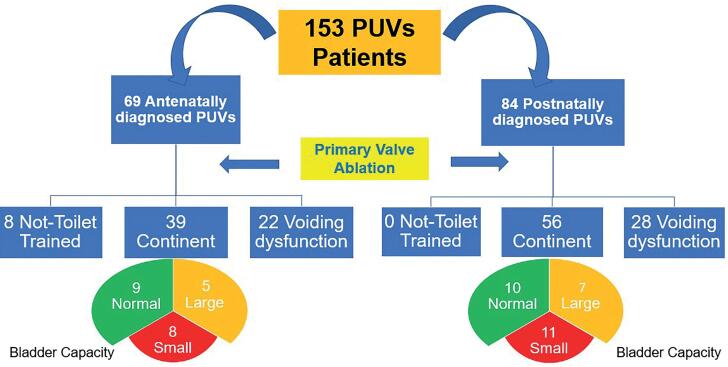
Flow chart of our study groups with bladder function and bladder capacity outcome.

Bladder function in the whole study groups is shown in Figure-1. In group 1, 8 patients were not toilet trained at last follow-up, while continence was achieved in 39 out of 61 boys (64%) and voiding dysfunction was noticed in 22 boys (36%). In group 2, continence was achieved in 56 patients (66.5%) while voiding dysfunction persisted in 28 (33.5%). Comparison between the two groups showed no difference in the bladder function outcome as shown in Table-1.

**Table 1:** Comparison of the patients’ outcome in both groups.

Variable		Group 1 N= 69	Group 2 N= 84	P Value
**Mean Age at Valve Ablation**				
	Median (IQR)	10 days (6–18)	7 months (3–19)	<0.001*
**Follow-up period**				
	Median (IQR)	6.5 (3–9)	7 (3.5-9)	0.41
**Chronic Kidney Disease (CKD)**				
	No. (%)	15 (22%)	31 (37%)	0.04*
**Bladder Function**				0.73
	Continence, No. (%)	39 (64%)	56 (66.5%)	
	Voiding Dysfunction, No. (%)	22 (36%)	28 (33.5%)	

* Significant

P value was calculated using Mann Whitney and Chi Square tests.

Management of voiding dysfunction included timed and double voiding, biofeedback, and medical treatment including oral anticholinergics, desmopressin, and alpha blockers. Some children required clean intermittent catheterization (CIC) and/or overnight catheter drainage to achieve continence and to protect the upper tract; this was the case in 3 children from group 1 and 4 from group 2. None of our patients needed intestinal bladder augmentation; however, 3 patients (1 in group 1 and 2 in group 2) underwent uretero-cystoplasty.

Urodynamic study was carried out for 50 patients: 22 patients from group 1 and 28 patients from group 2. Mean age at time of urodynamic examination was 8 years (range: 3-14) for group1 and 7.5 years (range: 4-16) in group 2. While Q-max, percent bladder capacity and PVR were comparable in both groups (13 vs. 11.6, 280 vs. 274 and 64 vs. 99), percent PVR was significantly higher in group 2 than group 1 (3.27 vs. 1.44, p <0.001). Detrusor overactivity was found in 13 children in group 1 while it was observed in 18 children in group 2 and the difference was not statistically significant (p 0.70). Comparison of urodynamic parameters of both groups revealed no significant difference except for the ratio of PVR/15% capacity PVR. Table-2 demonstrates the urodynamic parameters of both groups and the difference between them and the corresponding p values.

**Table 2:** Comparison of urodynamic parameters between the two groups.

Variable		Group 1 (AND) No. = 22	Group 2 (PND) No. = 28	P Value
**Bladder Capacity (mL)**				
	Mean ± SD (Range)	280 ± 110 (115-480)	274 + 152 (80-800)	0.87
**Percent Capacity**#				
	Mean ± SD (Range)	1.010 ± 0.46 (0.27-1.78)	1.004+0.52 (0.38-2.96)	0.96
**Compliance (mL/cmH2O)**				
	Mean ± SD (Range)	17.87 ± 9 (8-40)	16.23 + 6.66 (6-28)	0.45
**Detrusor Overactivity**				
	Yes	13 (59%)	18 (65%)	0.07
	No	9 (41%)	10 (35%)	
**Postvoid Residual (PVR)**				
	Mean ± SD (Range)	64 ± 75 (10-300)	99 + 116 (10-620)	0.21
**Percent PVR**				
	(PVR/15%Capacity)	1.44 ± 1.22 (0.35-4.44)	3.27 ± 2.11 (0.40-7.75)	0.002*
**Q Max (mL/s)**				
	Mean ± SD (Range)	13 ± 4.7 (6-25)	11.6 ± 4.4 (5-22)	0.24

# Capacity/Expected Capacity

* Significant

AND, antenatal diagnosis; PND; postnatal diagnosis; SD, standard deviation

P value is calculated using Chi Square and Levene's independent sample tests.

## DISCUSSION

Bladder outlet obstruction caused by PUVs in early life leads to a mixture of structural, functional, and biochemical changes that will judge the evolution of the bladder function later in a child's life and may persist even after the relief of obstruction (6, 7, 20). Valve bladders are usually thick with hypertrophied muscles, increased type III collagen fibers and deranged myosin and elastin ratios, leading to an impaired contractile strength and poor compliance with ultimately high filling pressures (7, 20). Bladder dysfunction has been related to poor renal function outcome in PUVs patients, and for that reason it was reasonable to assume that early detection and management of bladder obstruction will help bladder recruitment and eventually improve bladder function and the ultimate renal function in those patients.

Age at time of diagnosis might be a potential predictor for functional outcome after valve ablation (2, 3). Controversies exist over the last two decades, such as an early presentation of valve is more deleterious or children undergoing early intervention may show worse outcome or vice versa (2, 3, 21–23). Early presentation was viewed as a poor prognostic factor and suggestive of a serious obstruction, whereas late presentation was presumed to represent less obstruction with modest clinical significance and a more satisfactory outcome. Other researchers have also shown a poor outcome of late presentation. Some centers, where a standard antenatal ultrasound is performed with emphasis on renal and bladder evaluation, have primarily antenatal diagnosis, while others, where the facility for a comprehensive antenatal care is lacking rely on postnatal diagnosis of PUVs. Accordingly, in this study, median age at time of valve ablation is different in both groups. It was 10 days in group 1 and 7 months in group 2.

Meanwhile, data regarding the influence of timing of diagnosis were conflicting. While antenatal detection was thought to improve the renal outcome of PUVs (2, 3), earlier studies failed to show that long-term outcome of boys with antenatally detected PUV is better than of symptomatic boys with postnatally diagnosed disease (21, 22). In our series, we proved that early diagnosis and hence early valve ablation in the neonatal period improves future renal function and protects against the development of CKD. The difference in renal function outcome was significantly better in the prenatally detected group when compared to the postnatal group.

Delayed diagnosis and treatment are certainly a risk factor for renal impairment, a finding supported by many. Ansari et al. have found serum creatinine to differ significantly among two groups of children below and above the age of 2 years, for whom primary valve ablation was performed (23). Bajpai et al. reported a similar observation where they noted that recovery of renal function was lowest in older children where diagnosis was delayed in a cohort of 58 children (24). Sarhan et al. (3) proved that the potential to recover the renal function is believed to be significant in patients in whom early detection and early intervention of PUVs was performed.

Because of the contradictory results regarding the effect of high diversion and vesicostomy on the future bladder function in PUVs patients, we only included cases who underwent primary valve ablation in our study. Early valve ablation was advocated in PUVs patients as it allows for early bladder cycling and produces a better bladder compliance and function than delayed ablation or diversion (15). We could not find any advantage of early versus delayed valve ablation in terms of bladder function in our study, and the outcome was comparable between the two groups. It is clear from our results that all urodynamic parameters were better with early valve ablation as compared to delayed fulguration, although the difference was not statistically significant. The incidence of voiding dysfunction in our study cases was also similar in the two groups.

Lower urinary tract dysfunction is a frequent consequence in PUVs patients. While Bauer et al. have reported 5 types of urodynamic patterns including normal, uninhibited, small capacity bladder, high voiding pressure and myogenic failure (25), Peters et al. have described 3 characteristic patterns: myogenic failure with overflow incontinence (40%), detrusor hyperreflexia (29%) and bladder hypertonia (31%) (7). In another study by Misseri et al., myogenic failure was reported in 5.9% of children. The authors mentioned they used the same definition of myogenic failure as postulated by Peters et al. and explained the difference in incidence to aggressive management and follow-up of their patients (26). Myogenic failure was documented during follow-up in 4 patients in each group of our patients. Bladder hypo contractility was expected in patients after PUVs ablation, and the incidence increases with time (12, 27). Myogenic failure has been linked with hypertrophied bladder neck and poor bladder sensibility (28).

We also studied urinary continence among our cases, and the incidence was nearly equal in both groups. The achievement of day and night continence in PUVs patients has been documented to be significantly delayed than their healthy peers (14). They reported a 68% continence rate in PUVs patients versus 99% in healthy controls at 6 years. We documented a similar continence rate among our patients after a median follow-up of 6.5 years. The development of urinary continence in PUVs patients has been proven to be slow and improves overtime. Many studies investigated the rate of continence in children with PUVs and different continence rates have been reported at different ages (2, 4, 5, 29). Sarhan et al. (2) studied 65 prenatally detected PUVs patients and found a 49% of them continent at a mean age of 3 years. Parkhouse et al. had a 55% rate of continence by the age of 5 years, but all achieved continence at puberty (5). Lal et al. followed patients for a longer period and found the continence rate increased from 54% below the age of 12 years to 71% from 12 to 18 years and reached 91% after the age of 18 years (29).

Bladder capacity in the prenatal group was slightly larger than in the postnatal group. Perhaps median age at urodynamic testing was different and hence we calculated the normal expected capacity for age what was expressed as percent capacity. Using this proportion, children in group 1 had larger capacity than those of group 2. The same applied to compliance. Hypercontractility and poor compliance usually subsides after valve ablation and high voiding pressures gradually decreases within 1-3 years (11, 27). In addition, after valve ablation, the mean bladder capacity has been reported to be significantly bigger than the expected age adjusted normal volume (11).

The incidence of detrusor overactivity was also different in our study. Children from group 2 had more overactivity (65% in group 2 vs. 59% in group 1). Emir and associates have found 5 patients out of 26 studied with low compliance and small bladder capacity on urodynamics, who required later bladder augmentation (30). All of them were treated by primary valve fulguration after 2.5 years. They concluded that early relief of infravesical obstruction could have an improving effect on bladder function. Among our patients, 3 patients needed augmentation uretero-cystoplasty, and they improved on follow-up.

The absolute PVR difference was not statistically significant between the two groups. Mean PVR was 64mL and 99mL in groups 1 and 2, respectively. When PVR was related to 15% of the expected capacity for age; a variable we called percent PVR; the difference between the groups was statistically significant. Our explanation was that this finding may result from long- standing obstruction and overstretch of the bladder wall. A significant PVR after valve ablation was documented in about 50-70% of patients despite high voiding pressures (11). This residual urine even increased when patients reach puberty and learn how to empty their bladder better to avoid complications (13).

We are aware of the limitations of this retrospective study. The two groups are not properly controlled and probably the lack of uniform data from voiding cystometry that rendered free uroflowmetry the only voiding data we had. We tried our best to standardize the urodynamic measurement, nevertheless, our best might not have been enough. Delicate details in performing the test may mount to clinical differences. We also did not study the effect of bladder dysfunction on the ultimate renal function. In addition, the timing of urodynamic study was late and by this time the bladder ultra-structure changes become fixed and almost turn out be irreversible. This would have created a major bias in both the groups and can be one important reason for not finding any difference with statistical significance in two groups.

Finally, in the current study we tried to compare the effect of early prenatal diagnosis and hence intervention in children with PUVs against late diagnosis and management in terms of bladder function in a homogenous group of patients who underwent primary valve ablation and observation. The previous studies included patients who underwent urinary diversion (vesicostomy-ureterostomy) together with valve ablation, which might be a source of bias.

## CONCLUSIONS

Primary ablation of PUVs during the early neonatal life possibly provides the optimum chance of children with PUVs to obtain the best possible renal function. Despite renal function is significantly better when PUVs were diagnosed antenatally, bladder function outcome in those cases was not significantly better than in late presenting cases. Future prospective studies evaluating the impact of early valve ablation on the future bladder function in PUVs patients are encouraged.

## ABBREVIATIONS

PUV: Posterior Urethral Valve
VCUG: Voiding Cystourethrogram
PVR: Post Void Residual Urine
GFR: Glomerular Filtration Rate
CKD: Chronic Kidney Disease
ESRD: End Stage Renal Disease
UTI: Urinary Tract Infection
CIC: Clean Intermittent Catheterization
